# Using Fluorescence Intensity of Enhanced Green Fluorescent Protein to Quantify *Pseudomonas aeruginosa*

**DOI:** 10.3390/chemosensors6020021

**Published:** 2018-05-03

**Authors:** Erin Wilson, Macduff Okuom, Nathan Kyes, Dylan Mayfield, Christina Wilson, Derek Sabatka, Jasmin Sandoval, Jared R. Foote, Michael J. Kangas, Andrea E. Holmes, Arin L. Sutlief

**Affiliations:** 1 Department of Chemistry, Westminster College, New Wilmington, PA 16172, USA, wilsonee@westminster.edu; 2 Departments of Chemistry, Doane University, Crete, NE 68333, USA

**Keywords:** *Pseudomonas aeruginosa*, enhanced green fluorescent protein, fluorescence spectroscopy, bacterial quantification, biofilms, fluorescent probe

## Abstract

A variety of direct and indirect methods have been used to quantify planktonic and biofilm bacterial cells. Direct counting methods to determine the total number of cells include plate counts, microscopic cell counts, Coulter cell counting, flow cytometry, and fluorescence microscopy. However, indirect methods are often used to supplement direct cell counting, as they are often more convenient, less time-consuming, and require less material, while providing a number that can be related to the direct cell count. Herein, an indirect method is presented that uses fluorescence emission intensity as a proxy marker for studying bacterial accumulation. A clinical strain of *Pseudomonas aeruginosa* was genetically modified to express a green fluorescent protein (PA14/EGFP). The fluorescence intensity of EGFP in live cells was used as an indirect measure of live cell density, and was compared with the traditional cell counting methods of optical density (OD_600_) and plate counting (colony-forming units (CFUs)). While both OD_600_ and CFUs are well-established methods, the use of fluorescence spectroscopy to quantify bacteria is less common. This study demonstrates that EGFP intensity is a convenient reporter for bacterial quantification. In addition, we demonstrate the potential for fluorescence spectroscopy to be used to measure the quantity of PA14/EGFP biofilms, which have important human health implications due to their antimicrobial resistance. Therefore, fluorescence spectroscopy could serve as an alternative or complementary quick assay to quantify bacteria in planktonic cultures and biofilms.

## 1. Introduction

The goal of this study is to investigate the potential of fluorescence spectroscopy to: (1) quantify genetically engineered *Pseudomonas aeruginosa* (PA14/EGFP) through the detection of the enhanced green fluorescent protein (EGFP) and to (2) correlate fluorescence to plate counting colony-forming units (CFUs), which is the gold standard of bacterial quantification.

*Pseudomonas aeruginosa* (PA) is a common Gram-negative bacterium that is responsible for more than 10% of all hospital acquired infections [1]. PA is prevalent in soil, water, and the human skin flora, and is virtually impossible to prevent exposure in natural environments [2,3]. While this bacterium is typically found on the surface of the body, it is an opportunistic pathogen that can form antibiotic-resistant biofilms, causing a wide array of infections [2,4–8], such as skin (particularly in burn patients), urinary tract, kidney, and surgical site infections, as well as pneumonia and sepsis [5,9,10]. These developing biofilms are also strongly associated with respiratory infections in cystic fibrosis patients, and account for a majority of the morbidity and mortality for this disease [3,11]. These infections are not only persistent, they are also severe when compared with many other bacterial infections [1].

A biofilm is a complex, three-dimensional microbial community that grows on a surface and interacts with the surrounding environment [12]. They contain live and dead bacterial cells of one or more species, as well as extracellular polymeric substances and other materials that are secreted by the cells [13]. While biofilms are problematic in medical and industrial settings, they can also be beneficial and used in waste remediation and other applications [6,13,14].

Many different modalities have been established to quantify bacteria in planktonic and biofilm cultures, including direct cell enumeration (plate counting, microscopy, Coulter counting, and flow cytometry) [15–25], optical density [26–28], dry mass analysis [29–31], protein assays [32–38], and several staining methods [16,22,23]. The current gold standard method to quantify bacteria is cell enumeration by plate counting, offering multiple advantages. It is scalable to any culture density through serial dilution prior to plating. It also counts only the cells that are capable of growing into a colony on a plate, which are termed viable cells [39]. However, plate counting could lead to inaccurate quantification if a clump of cells forms a single colony and negates the one-cell, one-colony assumptions. Plating counting is also limited by the long incubation periods that are required and the technical demands of the techniques that are needed for accurate results. Other disadvantages to plate counting are the time it takes and the technical demands of the technique for good results. Another very common method of cell density estimation is OD_600_, or optical density. OD_600_ is used as the optical standard method for cell-density quantification where 600-nm wavelengths are scattered by the bacterial cells. OD_600_ is correlated by a bacterium-specific calibration curve to culture density. This technique has the advantage of being very rapid and simple, and requiring little expertise. Its chief disadvantage is the inability to distinguish between live and dead cells.

Therefore, the use of a fluorescent reporter such as EGFP may represent a good solution because it is quick and simple, yet only live cells will be measured, as dead cells quickly cease to fluoresce [40]. Another potential advantage of this method for biofilm research is that a variety of genetically modified fluorescent proteins have been developed with different emission wavelengths across the visible spectrum [41]. Biofilms are very diverse, and can consist of multiple species. Therefore, the ability to differentiate between species, such as bacteria that are genetically modified to express fluorescent proteins of a variety of emission colors, would be beneficial. This assists in tracking the growth of a single species within a complex environment in order to better understand bacterial cell interactions in mixed-species biofilms [42–44]. EGFP is a mutant of green fluorescent protein (GFP), and was used in this study due to its higher fluorescence properties [41,45,46]. Therefore, EGFP was used as the fluorescent reporter for PA14, because the literature demonstrates that GFP does not interfere with cell growth and function. Chalfie et al. reported that GFP-tagged *E. coli* cells grew well and continuously without having suffered any toxic effects in comparison to the control bacteria [20]. Other reports substantiate this claim [21,27,47].

In this study, the quantification of PA14/EGFP by EGFP fluorescence, plate counting, and optical density were carried out in parallel to evaluate the potential usefulness of EGFP fluorescence as a fast and simple bacterial quantification method for bacterial research studies, such as biofilm attachment and antibacterial surfaces. This study of quantification by fluorescence was further confirmed independently using the same PA14/EFGP. Important considerations and limitations are discussed. Furthermore, the fluorescence of liquid cultures derived from PA14/EGFP biofilms were demonstrated to establish the potential to extend this technique to the quantification of biofilms.

## 2. Materials and Methods

### 2.1. FAB Minimal Media Preparation

Fastidious anaerobe broth (FAB) minimal media was prepared under sterile conditions. For 1 L of FAB media, 2 g of (NH_4_)_2_SO_4_, 6 g of Na_2_HPO_4_, 3 g of KH_2_PO_2_, 3.0 g of NaCl, 0.093 g of MgCl_2_, and 0.011 g of CaCl_2_ were dissolved into deionized water. The pH was adjusted to 6.8–7.0. The solution was autoclaved and stored for six months at room temperature, or one year at 4 °C. After autoclaving and cooling, 1 mL of filter sterilized trace metals solution and sodium citrate solution (300 mM) were added.

To make 100 mL of the sodium citrate solution, 7.74 g of trisodium citrate (Na_3_C_6_H_5_O_7_*·*H_2_O) was dissolved into deionized water. The solution was sterilized using a 0.22-μm filter and stored at 4 °C. The trace metal solution was made up of 200 mg/L of CaSO_4_, 200 mg/L of FeSO_4_, 20 mg/L of MnSO_4_, 20 mg/L of CuSO_4_, 20 mg/L of ZnSO_4_, 10 mg/L of CoSO_4_, 10 mg/L of Na_2_MoO_4_, and 5 mg/L of H_3_BO_3_ in deionized water. The pH was adjusted to make the solution acidic in order to prevent trace metals from precipitating. A 0.22-μm filter was used to sterilize the trace metals solution.

### 2.2. M9 Minimal Media Preparation

To make 1 L of M9 minimal media, 200 mL of sterile M9 salt solution, 2 mL of sterile 1 M MgSO_4_, 100 μL of sterile 1 M CaCl_2_, and 20 mL of sterile 20% (*w*/*v*) glucose solution were dissolved into sterile deionized water at a final volume of 1 L. One liter of M9 salt solution was prepared using 64 g/L Na_2_HPO_4_ · H_2_O, 15 g/L KH_2_PO_4_, 2.5 g/L NaCl, and 5.0 g/L NH_4_Cl in deionized water. Separately, 1 M MgSO_4_ and 1 M CaCl_2_ solutions were made. These three solutions were sterilized by autoclaving. A 20% glucose solution was made and sterilized by a 0.22-μm filter.

### 2.3. PA14/EGFP Planktonic and Biofilm Culture—Procedure 1

**Figure 1.**
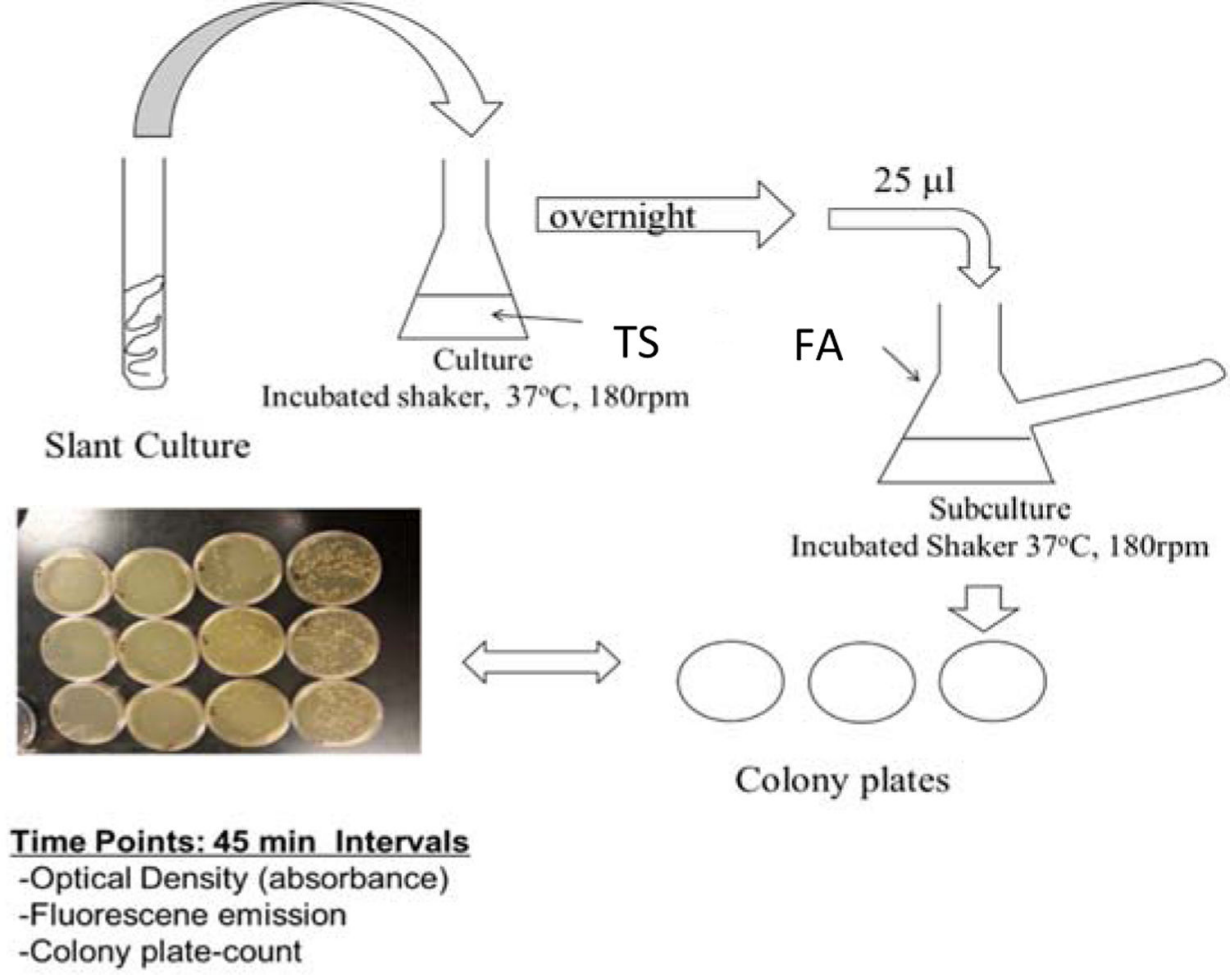
Flow chart for the preparation of the PA14/EGFP culture. From the slant, a swipe of the culture was introduced into 25 mL of tryptic soy broth (TSB) media and incubated at 37 °C on a shaker for 12 h. Then, 25 μL of the culture was added to a side-arm flask with 25 mL of fastidious anaerobe broth (FAB) media and incubated for another 2 h or until an OD_600_ of 0.03 was obtained. Absorbance readings were taken, and samples were prepared for assaying.

The genetic modification of PA14/EGFP was performed at the Helmholtz Institute for Infectious Disease Research by construction plasmids, which constitutively express the bright mutant of GFP [48]. Cultures were maintained for short term at room temperature on tryptic soy agar (TSA) slants or plates. Prior to planktonic or biofilm studies, 25 μL of an overnight culture of PA14 (overnight culture conditions of 37 °C, 180 rpm, minimum of 12 h in tryptic soy broth (TSB) media) was diluted to 25 mL with FAB media, and this subculture was incubated at 37 °C while shaking at 180 rpm for another 2 h or until an optimal concentration (OD = 0.030) had been obtained (Figure 1). While TSB media is a common media used to grow PA14, TSB media is fluorescent itself due to the presence of tryptic soy proteins. In order to avoid autofluorescence contribution from the media, PA14 was diluted with excess minimal media that does not contain fluorescent compounds. Planktonic PA14/EGFP studies continued with this subculture under the same conditions, and samples were collected at 45-min time intervals for quantification by optical density, fluorescence, and cell colony plating. For biofilm formation studies, the biofilms were grown in a drip flow reactor according to the protocol of Goeres et al. [49]. A 25-μL sample of subculture was added to sterile glass slides submerged in 15-mL FAB minimal media for surface inoculation at 37 °C over a six-hour time period with no shaking. The inoculated slides were then placed in the drip-flow reactor channels and incubated for 30 h at 37 °C with a constant FAB minimal media flow of 1.0 mL/min. The biofilms were scraped into 50 mL of sterile FAB minimal media and homogenized into suspension. Biosafety level 2 practices were used to handle PA14/EGFP [50].

### 2.4. PA14/EGFP Planktonic and Biofilm Culture Confirmation Study—Procedure 2

This procedure was used in collecting the data shown in Section 3.2 (independent protocol performed at Westminster College using the Miles and Misra method to confirm the relationship between PA14/EGFP cell count and fluorescence). Cultures were maintained for short term at room temperature on TSA plates. Prior to quantification by plate counting or fluorescence, an overnight culture of PA14 (overnight culture conditions of 37 °C, 180 rpm, minimum of 12 h in TSB) was prepared. The culture was centrifuged at 5000 rpm for one minute, and the TSB was poured off and replaced with M9 minimal media for quantification. The culture was resuspended with gentle agitation. Serial 1:2 dilutions were performed to generate cultures with different cell densities. Final cultures were quantified by fluorescence, as described below. They were also quantified by plate counting using the Miles and Misra method [51]. Plates were divided into eight sections. Serial dilutions of the bacterial cultures were plated in 20-μL aliquots in each section of the plate, and counted after overnight incubation at 37 °C.

### 2.5. Assays Procedure

Optical density was determined with a Spectronic 21 UV-VIS spectrophotometer at wavelength 600 nm in a side-arm flask with a blank of sterile media. Fluorescence intensity was determined for 2 mL of culture obtained at each time point with either a Shimadzu RF 51301PC or a Perkin Elmer LS-55 fluorophotometer. An excitation wavelength of 475 nm was used, and an emission wavelength of 514 nm was monitored at room temperature under constant stirring to avoid the settling of the bacterial cells to the bottom of the cuvette. Cell enumeration was performed according to standard plate enumeration methods [52]. This was done by diluting 100 μL of the suspended culture in 10 mL of media and serial diluting the culture, plating the culture dilutions, culturing the plates at 37 °C overnight and manually counting the number of colonies formed. Using this count and the known dilution factor, the total cell count of the original culture was determined.

### 2.6. Data Analysis

**Figure 2.**
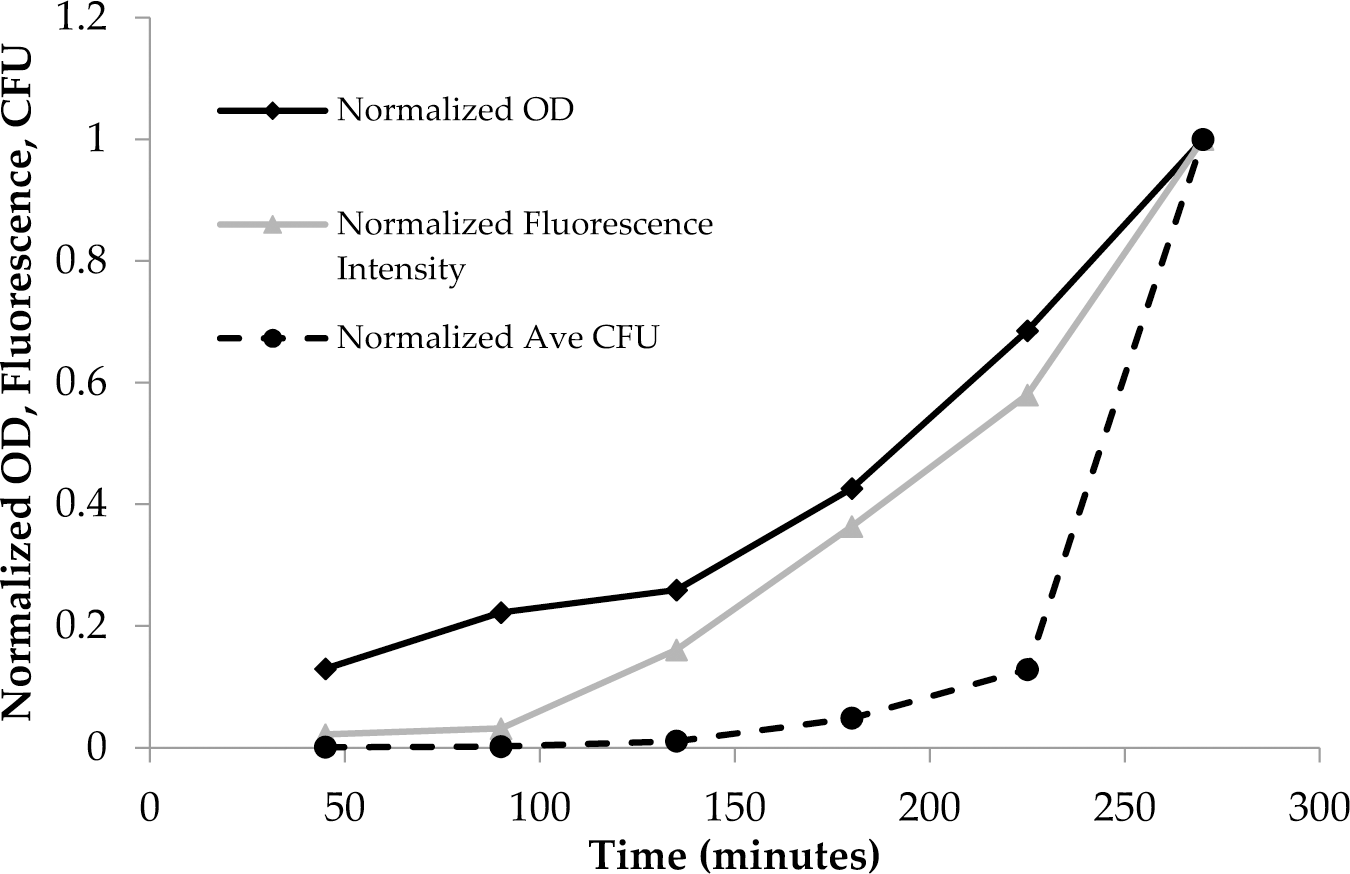
Normalized time dependent optical density (OD_600_), fluorescence emission at λ_ex_ = 475 nm, and the average plate count of colony-forming units (CFU). The normalized units increased as a function of time due to the increase in bacterial population for OD_600_, fluorescence, and CFUs.

In Figure 2, data were analyzed using Microsoft Excel and normalized between 0 and 1 to show all of the data in one graph to demonstrate the interrelationship between OD_600_, fluorescence, and colony-forming units. Curve fitting was carried out by Igor software.

## 3. Results

### 3.1. Fluorescence Spectroscopy as a Tool to Quantify the Planktonic Bacteria of PA14/EFGP

PA14/EGFP living cells are fluorescent and display an emission profile characterized by a maximum at 514 nm when excited with visible light (475–495 nm) [20]. As Table 1 shows, the absorbance, fluorescence, and plate count increased with time. This trend is more clearly shown in Figure 2, where the data from Table 1 were normalized to the largest observed value in each assay so that OD_600_, fluorescence, and CFU values were between 0 and 1. The normalized OD_600_ and fluorescence intensity increased from 45 min to 270 min, and accordingly, the normalized number of colonies increased as well.

**Figure 3.**
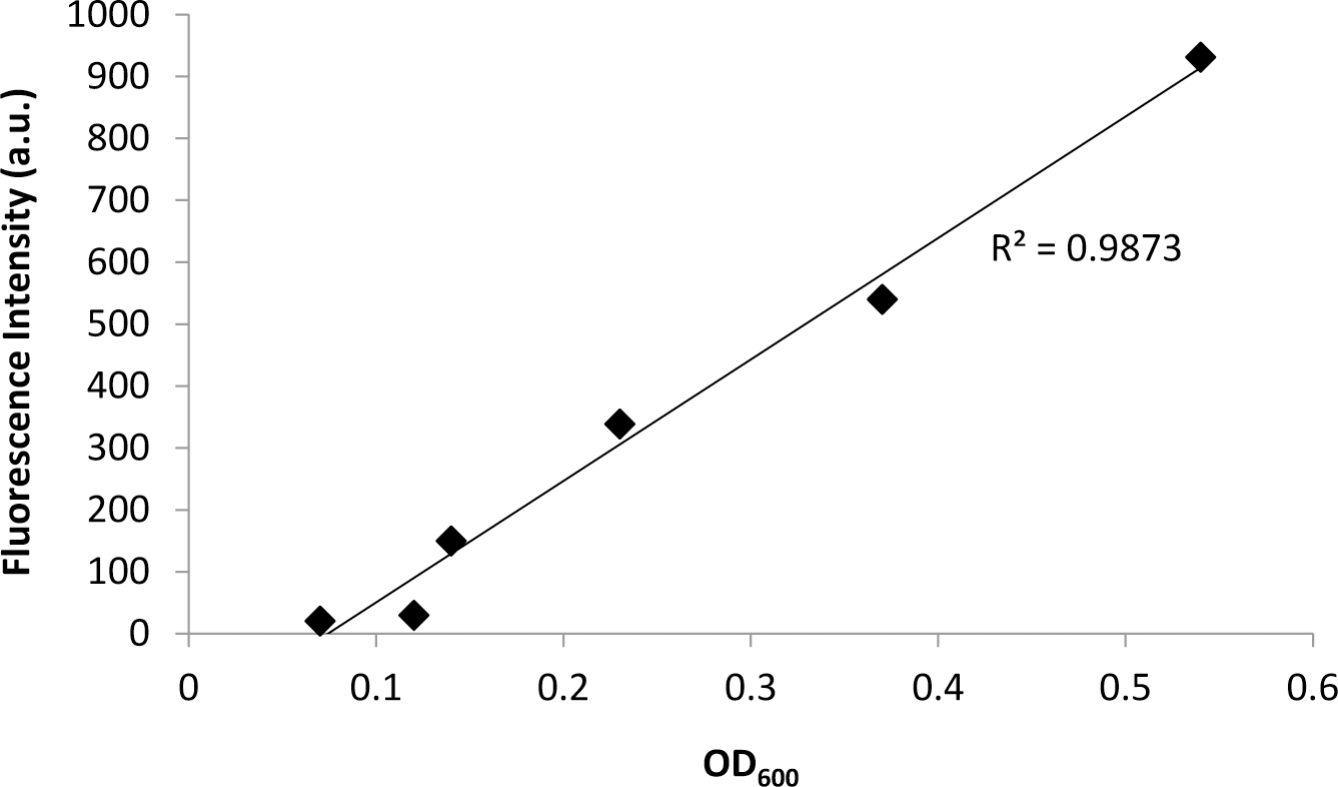
Fluorescence emission and absorbance of PA14/EGFP shows a linear relationship with R^2^ = 0.9873. The fluorescence increases from 0 a.u. to 931 a.u. with an increasing OD_600_ ranging from 0.07 to 0.5.

Bacterial growth was observed to exhibit four distinct phases: the lag phase, the log phase or exponential phase, the stationary phase, and the death phase [47]. The log phase is the phase where cell doubling continues at a constant rate. In Figure 2, the fluorescence and OD_600_ curves show the log-phase growth curve between 45–270 min, where there is an exponential growth of the bacteria. The media alone did not show any fluorescence emission, as there are no fluorescent components in the media (data not shown). Furthermore, plotting the OD_600_ (x-axis) versus the fluorescence (y-axis) shows a linear correlation (Figure 3), with a correlation coefficient of R^2^ = 0.987. This demonstrates that the fluorescence emission of the EFGP is directly proportional to the optical density of the cell culture. The wavelength of 600 nm was chosen for OD, because 600 nm does not interfere with the absorbance of GFP, which absorbs at 475 nm. Therefore, relative errors are eliminated by avoiding interfering wavelengths between bacteria and fluorescent proteins or other fluorescent molecules that are present in PA, including pyocyanine and pyoverdine [53,54]. This point also strengthens the argument regarding why additional methods for bacterial quantification, such as fluorescence spectroscopy using EGFP, are desired, as they avoid the overlap of absorbing wavelengths.

**Figure 4.**
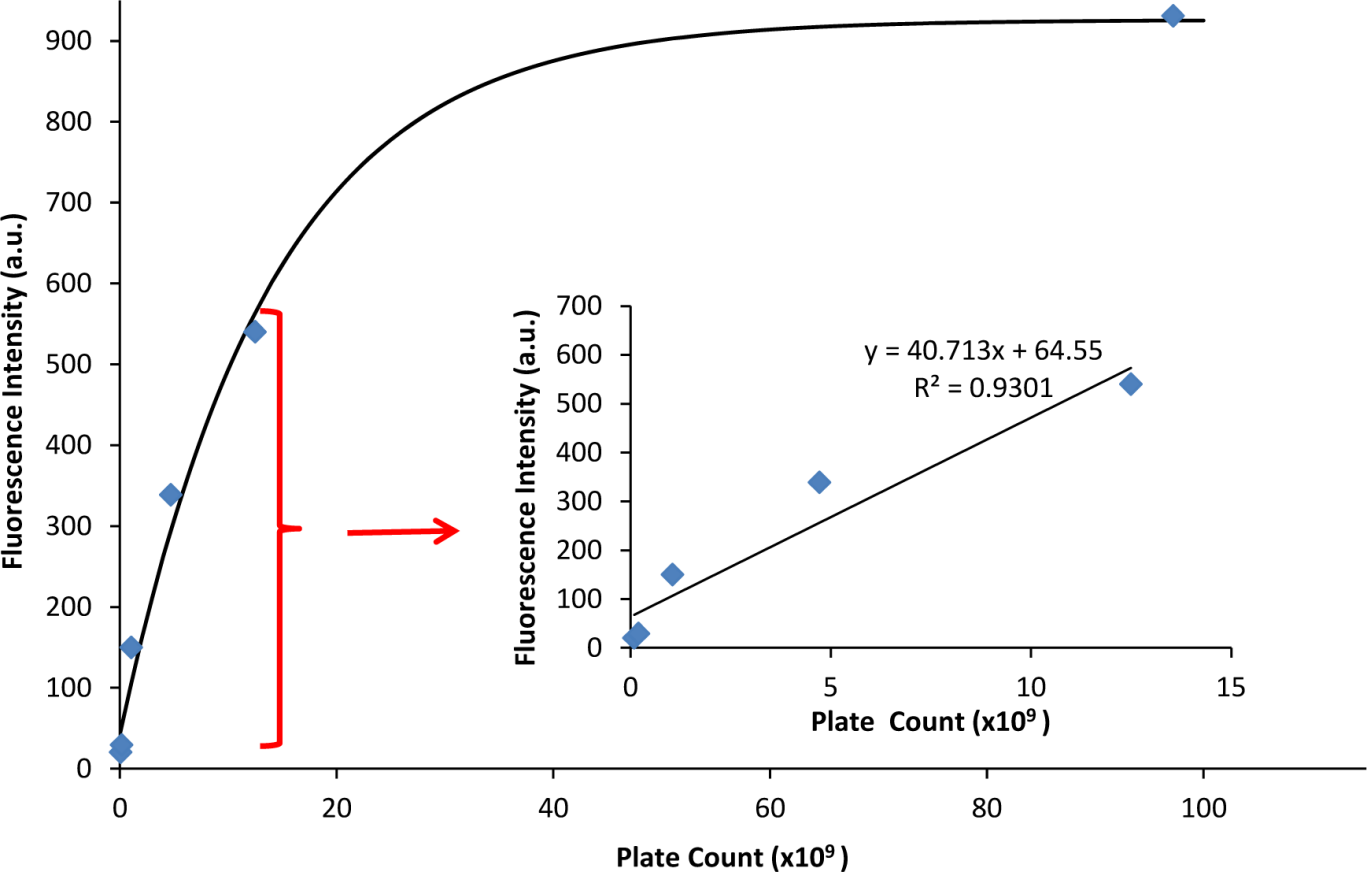
Fluorescence emission vs. plate count. The plate count (CFU) and the fluorescence of PA14/EFGP are related using an exponential fit calculated in Igor software: y = 926 *−* 884 exp(*−*x/14). The inset demonstrates a linear relationship between fluorescence emission and plate count at lower plate counts.

The fluorescence emission was graphed against the CFU/mL, and a linear relationship occurs at low values of fluorescence emission with an R^2^ value of 0.930 (Figure 4, inset). At higher emission intensities, the CFU values increased faster than the fluorescence intensity, causing a non-linear response (Figure 4). Therefore, a quantitative relationship between optical density and fluorescence emission can only be assessed during the logarithmic growing phase, because that is when the cells begin to grow exponentially, and chemical and physiological properties are the most uniform, such as the production of EGFP. It should be noted that this linear relationship is a calibration curve for PA14/EFGP only. Other strains of PA or other bacterial species would most likely yield a different calibration curve that would have to be obtained prior to any quantification experiments, especially if the stains are not tagged with a fluorophore similar to EFGP, and quantification relies on only the autofluorescence of NADH [55]. Thus, this method has limitations at high CFU counts, where fluorescence intensity may not correlate to a linear curve. Dilution of the samples may be required in order to lead to readings that are within the linear range.

### 3.2. Confirmation of Fluorescence Data

**Figure 5.**
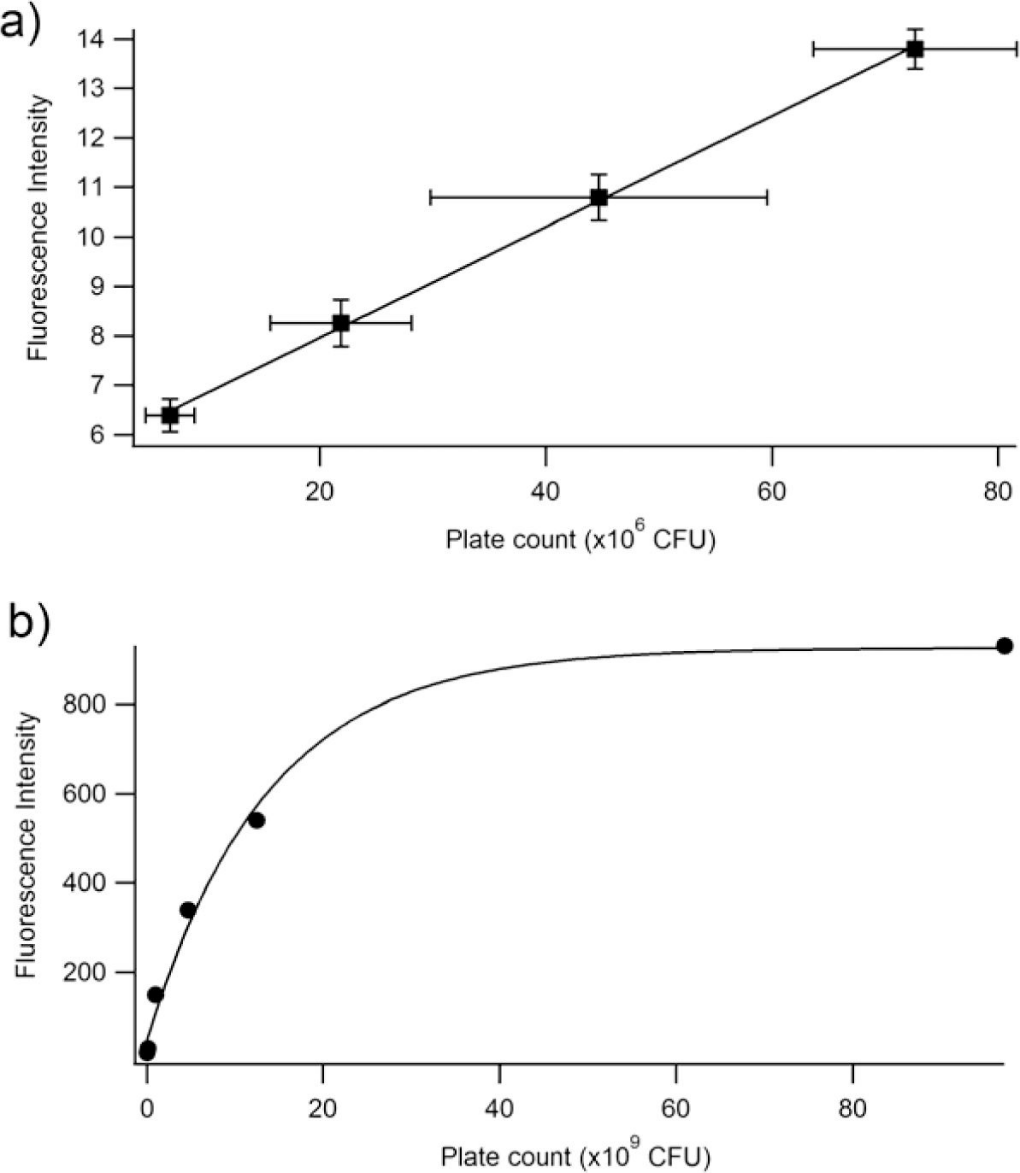
Fluorescence intensity at 514 nm vs. plate count (CFU) at two different ranges of CFU. **(a)** Linear range of fluorescence intensity vs. plate count relationship. The linear equation is y = 1.12 *×* 10*−7*x + 5.72. The R^2^ is 0.9992. (**b**) Full range of fluorescence intensity vs. plate count. The plate count (CFU) and the fluorescence of PA14/EFGP are related using an exponential fit: y = 5725 *−* 884exp(7.3 *×* 10^−11^x). These experiments were performed in triplicate, but due to small sizes, error bars are only shown in the focused linear range.

**Figure 6.**
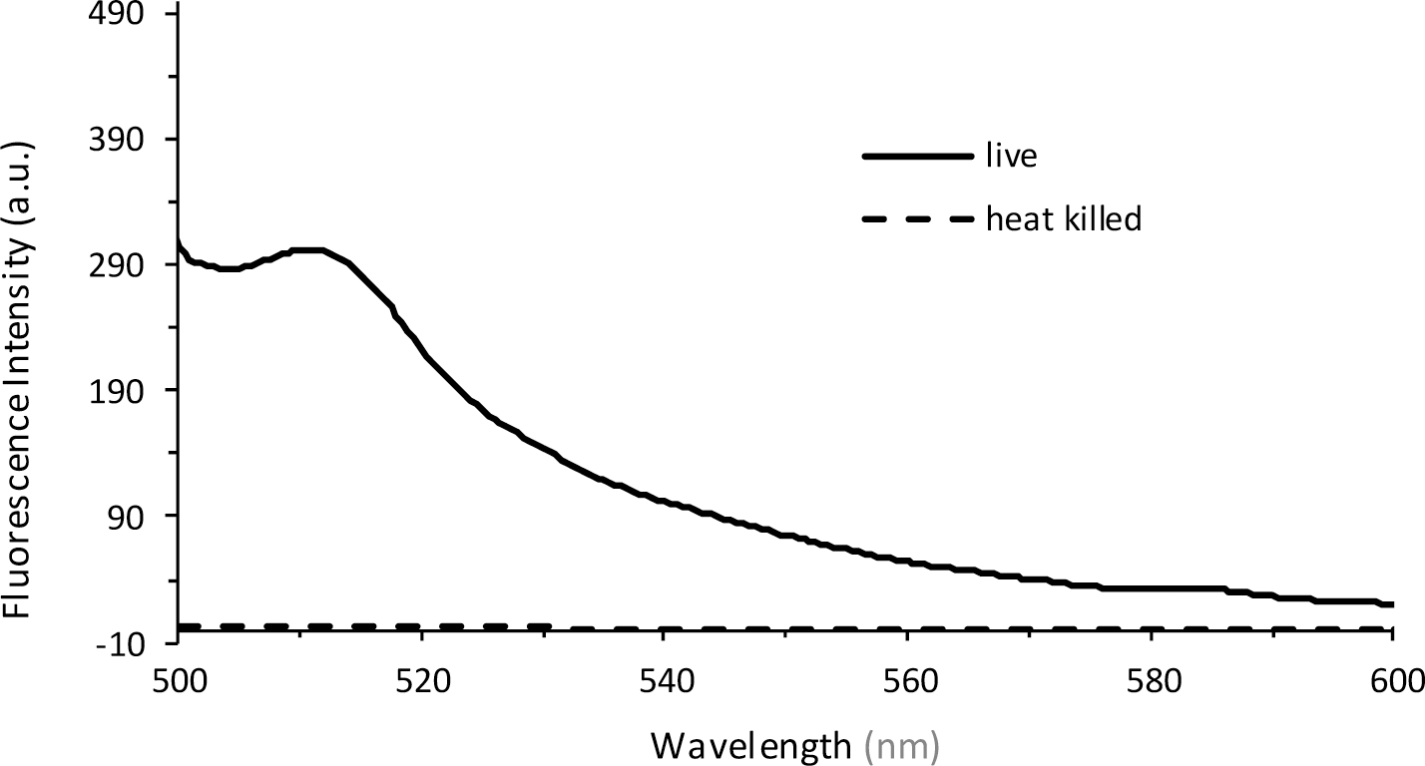
Fluorescence emission spectra recorded from live cells (solid line) and heat-killed cells (dashed line) at λ_Ex_ = 475 nm. The live PA14/EGFP cells were placed in a 60 °C water bath for 30 min, resulting in a heat-killed culture.

In order to determine whether the relationship between fluorescence and plate count is a reproducible and valid method for the quantification of bacteria, an independent study was performed at Westminster College using Procedure 2. The results confirm that at low plate counts, a linear relationship emerges between plate count between 10–80 (*×*10^6^) CFUs and fluorescence intensity, with a correlation coefficient of R = 0.999. The large error bars demonstrate the variation in plate counting, which is a typical challenge in microbiology and is encountered even under the best conditions. This is mostly because cell clumping can occur, and would no longer correlate to a ‘one cell to one colony’ count. Errors also occur in serial dilutions and subjective counting [52,56]. Further, fluorescence was determined for cell densities at 10^6^ CFUs. Lower concentrations could be determined if the instrumental settings were adjusted to increase the nanometer slit width in the excitation or emission window. Figure 5b continues to confirm the results seen in Figure 4, where at higher CFUs, the inner filtering effect causes an exponential fit rather than a linear relationship. Furthermore, experiments were performed to confirm that the fluorescence intensity observed corresponded to live cells only (Figure 6). The heat-killed cells did not demonstrate any fluorescence after cell death.

### 3.3. Fluorescence Spectroscopy for Quantification of PA14/EFGP Bacteria in Biofilms

**Figure 7.**
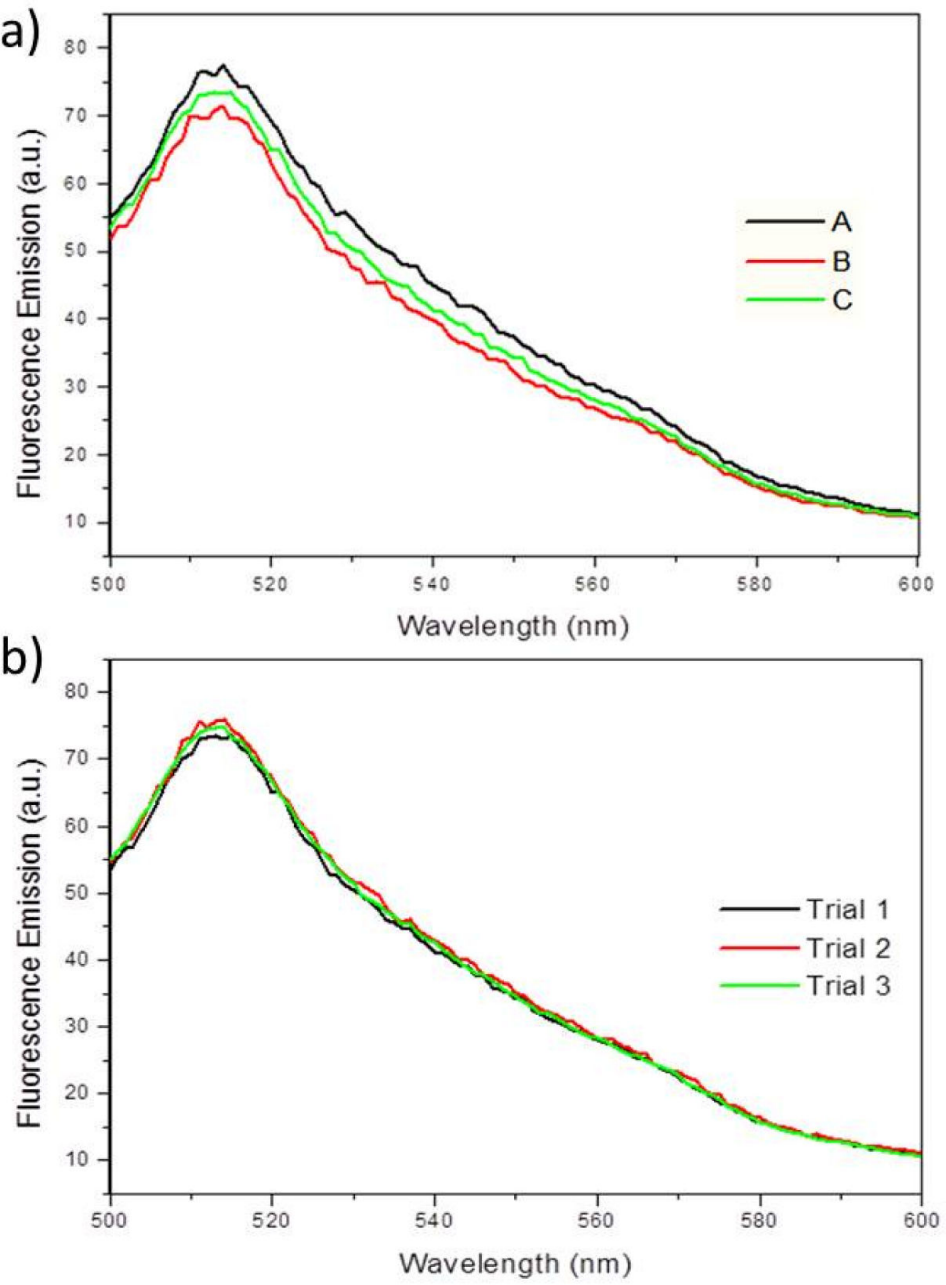
(**a**) Fluorescence emission spectra showing the fluorescence maxima at 514 nm, when excited at 475 nm for PA14/EGFP on three separate biofilms (A, B, C) grown in a drip-flow reactor. (**b**) Fluorescence emission spectra showing PA14/EGFP fluorescence maxima at 514 nm over three measurements (Trial 1, 2, and 3) on a single sample. No fluorescence quenching occurs during the replication of experiments within one sample.

To determine whether EGFP fluorescence can also be used to quantify biofilms, PA14/EGFP was grown in a drip-flow reactor, mimicking low shear conditions. As shown in Figure 7a, three biological replicates show average fluorescence emission intensities at 514 nm of 73 (*±*3) emission units. Multiple measurements for each replication concluded no loss of fluorescence intensity (Figure 7b), demonstrating that the EGFP is very stable and resistant to photobleaching. This indicates that the fluorescence spectroscopy of EGFP could serve as a chemical probe for planktonic bacterial cultures and bacterial biofilms. Since biofilm formation begins with planktonic, or free-swimming bacteria, which eventually adhere to a surface, fluorescence spectroscopy could be used to quantify cells before and after biofilm formation. Given that biofilms are usually very heterogeneous samples due to the uneven growth on a surface, it was expected that the fluorescence measurements would vary from biofilm to biofilm. However, since the fluorescence between samples was very similar, this method has the potential to be applied to quantifying biofilms.

## 4. Discussion

Viable cell enumeration through plate counting (CFUs) is a commonly used method of quantification for PA14/EGFP for determining the number of viable cells [24,25,57]. However, this technique may not be preferable in all situations, because it is time and labor-intensive, can require a lot of materials and supplies, and can take days for preparing cultures, streak plates, autoclaving, counting, etc., with enough replicates to obtain reproducible results [58]. Errors can commonly occur due to bacterial clumping and counting errors, where subjective bias by the researcher is a challenge, especially when the given number of colonies is high, and/or the count is done manually. Furthermore, cell enumeration methods do not differentiate between cell types, making the counting of specific species in a mixed culture impossible. If a fluorescence method could correlate to cell count, time, materials, and error could be reduced.

This study demonstrated that fluorescence spectroscopy can be used in dilute cultures to quantify PA14 bacteria when EGFP is used as a fluorescent tag. Fluorescence emission at 514 nm increased with increased bacterial growth when excited at 475 nm. A linear relationship (R^2^ = 0.999) between the absorbance and fluorescence of the bacteria was observed, indicating that fluorescence can be related to the viable bacterial cell count. At higher culture concentrations, the fluorescence curve displays non-linearity with increasing CFUs. This is most likely due to the inner filter effect, in which excitation light is depleted before penetrating the entire sample due to absorbance by highly concentrated chromophores [59]. This effect is exacerbated in this case due to the locally high concentration of the EGFP within the cells, and therefore, the samples would need to be diluted within the linear range for quantification.

In many ways, this method is similar to optical density as a method to quantify bacterial cell cultures. However, optical density has a disadvantage in that both live and dead cells are detected, making it difficult to determine the number of viable cells, which is usually the measurement of greatest interest. EGFP fluorescence occurs in live cells, but not dead ones, and is a property that has previously utilized in antibiotic-susceptibility assays [40]. Therefore, fluorescence may be a better measure of viable cell density than optical density. However, fluorescence quantification would require the genetic modification of the bacterial strain to express the EGFP or rely on the autofluorescence of NADH or other fluorescent components in the bacteria.

Finally, it was observed that PA14/EGFP biofilms can be grown and resuspended in solution fluoresce at 514 nm in a consistent manner. This was demonstrated by the ability to obtain similar fluorescence emission intensities at 514 nm in three separate PA14/EGFP biofilms, even in samples that are very heterogeneous. Therefore, this study demonstrated that new, more convenient assays could be developed for the characterization of biofilms instead of using more time-consuming techniques such as colony-forming unit counting.

The choice of which method to use for bacterial quantification is driven by what information is needed, equipment availability, cost, and ease-of-use. The assay described here could be easily implemented for bacterial growth, because fluorescence spectroscopy instruments are usually standard instrumental holdings in research and academic institutions. Furthermore, the assay is easy to set up and use, because it only requires the addition of fluorescence-modified bacteria to a fluorescence compatible cuvette followed by the emission measurement.

## 5. Conclusions

This study demonstrated the use of fluorescence spectroscopy for the quantification of PA14 tagged with EGFP in suspension and biofilm growth. This method showed an increase in fluorescence emission intensity with increasing cell numbers in correlation with established methods of absorbance and plated cell enumeration. It may also be useful for observing the growth of biofilms in a variety of treatment conditions. For example, fluorescence spectroscopy could be used for quantification before and after treatment, such as with antibiotics, when growing multiple fluorescently-tagged bacterial species in a single biofilm. However, the application of fluorescence spectroscopy with PA14/EGFP requires gene modification, and is therefore more usable in a biomedical research setting. If fluorescence spectroscopy were to be explored in a clinical setting, then the autofluorescent molecules in bacteria would have to be used for quantification, such as NADH or tryptophan [60]. In conclusion, the use of fluorescence emission intensity could be used as a quick and simple representation of changes in cell number over time in suspension and biofilm culture experiments.

## Figures and Tables

**Table 1.** Absorbance (OD readings), fluorescence emission at λ_ex_ 475 nm, and average plate count (CFU) from three plates.

Time (min)	Absorbance	Fluorescence	Ave Plate Count (CFU)
45	0.07	20.24	8.99 × 10^7^
90	0.12	29.48	1.96 × 10^8^
135	0.14	149.87	1.05 × 10^9^
180	0.23	338.66	4.72 × 10^9^
225	0.37	539.96	1.25 × 10^10^
270	0.54	931.05	9.72 × 10^10^
